# Association of miR-181c/d gene locus rs8108402 C/T polymorphism with susceptibility to Kawasaki disease in Chinese children

**DOI:** 10.3389/fped.2022.899779

**Published:** 2022-08-09

**Authors:** Meiqing Yao, Qin He, Manqiong Yang, Zhixiang Wu, Ying Li, Min Kong, Zhijuan Kang, Lu Yi, Yanan Hu, Lihua Huang, Zhuoying Li, Zuocheng Yang

**Affiliations:** ^1^Department of Pediatrics, Third Xiangya Hospital of Central South University, Changsha, China; ^2^Department of Pediatrics, Hunan Provincial People’s Hospital, Changsha, China; ^3^Center for Experimental Medicine, The Third Xiangya Hospital of Central South University, Changsha, China

**Keywords:** Kawasaki disease, miR-181, Nanos3, coronary artery lesions, single nucleotide polymorphisms

## Abstract

**Background:**

Kawasaki disease (KD) is an acute systemic vasculitis of unknown etiology. The rs8108402 C/T single nucleotide polymorphism (SNP) is located in the promoter region of miR-181-c/d gene and the intron of Nanos3 gene. The miR-181 family contributes to the pathogenesis of cardiovascular and inflammatory disorders, while Nanos3 is involved in DNA transcription regulation and cell proliferation. However, no studies have examined the association between miR-181c/d and Nanos3 polymorphisms and the susceptibility and progression of KD.

**Objective:**

The purpose of our study is to examine the association of miR-181c/miR-181d/Nanos3 gene locus rs8108402 C/T polymorphism with KD susceptibility, intravenous immunoglobulin (IVIG) responsiveness, and the development of coronary artery lesions (CAL).

**Methods:**

Peripheral blood specimens from 100 children with KD and 100 healthy children were collected. The polymorphism of rs8108402 C/T was detected using polymerase chain reaction-sequencing-based typing technique.

**Results:**

There were statistically significant differences in C and T allele frequency distributions between the KD group and healthy controls for the polymorphic site rs8108402 C/T (*P* = 0.002). The distribution of the genotypes CC, CT, and TT also presented statistical significant difference between the KD and control groups (*P* = 0.003). Compared to the rs8108402 C allele, the T allele was associated with increased KD susceptibility (OR = 2.080, 95% CI = 1.317∼3.283). However, there were no significant associations discovered between the rs8108402 C/T polymorphism and CAL formation or IVIG unresponsiveness in the study.

**Conclusion:**

SNP rs8108402 C/T located in the miR-181c/d promoter and Nanos3 intronic region is associated with susceptibility to Kawasaki disease but not with the development of coronary artery lesions or IVIG unresponsiveness in Chinese children.

## Introduction

Kawasaki disease (KD) is an acute febrile disease characterized by systemic inflammation of the small and medium-sized arteries that mainly affects children under 5 years of age. KD is the most common cause of acquired heart disease in childhood with its highly concerning sequelae of inflammation-induced coronary artery lesions (CAL) and associated complication outcomes including thrombosis, calcification, stenosis, and occlusion, with follow-up cases of adults who previously had KD reporting an increased lifetime risk of cardiac dysfunction and premature death. Prompt immunoglobulin administration is essential in KD management and significantly lowers CAL development risk. The etiology and pathogenesis of KD remain largely unknown. However, it is presumed that KD arises from infectious or environmental factor-triggered immune hyperactivation in genetically predisposed individuals. Surveys of possible KD-associated genetic loci or expression profiles are ongoing. Previous studies have revealed the associations of KD susceptibility with polymorphism loci in genes of the oxidative stress signaling pathway (1), inflammatory processes (2, 3), cell adhesion molecules (4), and microRNAs (5).

MicroRNAs (miRNAs) are endogenous, conserved, single-stranded non-coding RNAs of 21–25 nucleotides in length. The functional activities of miRNAs include the canonical pathway of mediating translational repression by binding to the 3′UTR of target mRNAs to induce mRNA degradation, as well as acting as mRNA decoys, activating Toll-like receptors, and directly enhancing intranuclear transcription. The miR-181 family of miRNAs are involved in various physiological and pathological processes including inflammation, immunity, cancer and apoptosis (6). The miR-181c and miR-181d genes of the miR-181 family are located as a cluster on chromosome 19 separated about 100 bp apart and have the same 5p seed regions but different 3p seed regions (6). MiR-181c is involved in a variety of cardiovascular diseases, including acute ischemic stroke, ventricular septal defects, and diabetic vascular complications. It also functions in the inflammatory response by negatively modulating the activation of CD4 + T cells and inhibiting NF-κB activation and downstream production of proinflammatory mediators such as TNF-α, IL-1β, and iNOS (7). While MiR-181d is associated with cardiovascular conditions including CVB3-induced myocarditis (8), tetralogy of Fallot (9), and maturation of DC cells (10). The NANOS3 gene, instrumental in growth, development, and transcriptional regulation, overlaps miR-181c and miR-181d gene loci and their respective promoter regions. Nanos3 inhibits Bax-dependent and -independent apoptotic pathways and may contribute to cancer invasion (11) and is essential for early embryos to protect the migrating primordial germ cells from apoptosis.

**FIGURE 1:**
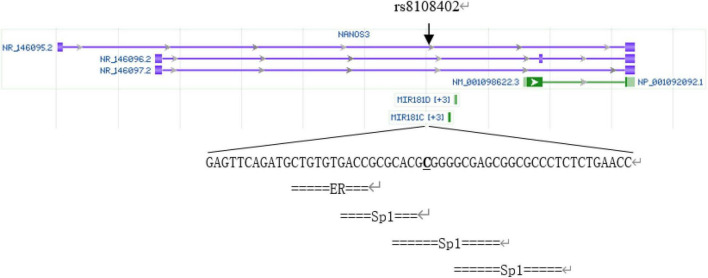
Bioinformatics analysis of SNP rs8108402.

The rs8108402 C/T polymorphism, located in the promoter 608 and 784 bp upstream of miR181c and miR181d precursor transcription start sites, respectively, and intron of Nanos3 gene 12,055 bp downstream of its transcription site, has been previously demonstrated to be associated with susceptibility to systemic lupus erythematosus in the Chinese population of Guanxi Province (12). We analyzed SNP rs8108402 using bioinformatics tools, as is shown in Figure 1. Transcription factor binding analysis shows that rs8108402 is the binding site of the middle one of three possible Sp1 transcription factors, indicating its importance in the control of expression of miR181c, miR181d, and possibly NANOS3 genes.^1^ Until now, the association between KD and the rs8108402 polymorphism has not been explored. Therefore we focused our study on the association between the miR-181c/d gene locus rs8108402 and KD susceptibility, IVIG responsiveness, and CAL risk.

## Materials and methods

### Patient population

The KD group consisted of 100 Han Chinese KD patients hospitalized at the Third Xiangya Hospital of Central South University between 2016 and 2019, all of whom met the diagnostic criteria of the Kawasaki Disease Research Committee of Japan (13). There were 58 males and 42 females in this group. The age distribution was between 2 months and 8 years of age, averaged at 2.78 ± 1.95 years. All members of the KD group were not treated with aspirin or intravenous immunoglobulin (IVIG) before admission. After admission, all KD group patients received IVIG treatment at the dosage of 2 g/kg. IVIG unresponsiveness was defined as presence of fever over 37.5°C 3 days after the initiation of IVIG treatment combined with the presence of at least one other KD symptoms. According to the criteria, 14 patients of the KD group were subclassified into the IVIG unresponsiveness group (KD-nIVIG), while 86 were included in the IVIG responsiveness group. The diagnostic indicators of CALs were coronary artery internal diameter > 3 mm for children < 5 years of age, or > 4 mm for those at or above 5 years of age; or the presence of a vessel segment with an internal diameter more than 1.5 times that of the adjacent segment or the presence of irregular coronary lumen as confirmed by echocardiography. According to the criteria, 25 patients in the KD group were subdivided into the coronary artery lesion group (KD-CAL), while 75 were divided into the no coronary artery lesion group (KD-nCAL).

The healthy control group, all of whom were healthy Han Chinese and were free from infectious, rheumatic, and cardiovascular diseases, or a previous history of KD, consisted of 100 children who underwent health examinations at the Health Management Center of the Third Xiangya Hospital of Central South University during the same time period. The control group consisted of 58 males and 42 females with an age distribution of 1.69 ± 2.05 years. There was no statistically significant difference between the age and gender distribution of the KD and control groups (*P* > 0.05).

Informed consent was obtained from the parents or legal guardians of all patients and healthy controls by written informed consent forms in advance. The study was approved by the Ethics Review Committee and Institutional Review Board of the Third Xiangya Hospital (No. 2020- S002).

### DNA extraction

A disposable blood collection tube containing the anticoagulant ethylenediaminetetraacetic acid dipotassium (EDTA-K2) was used to collect 2 ml of venous blood from children of the experimental and control groups. The blood specimens were placed in cryopreservation tubes and stored in a −80°C refrigerator. Blood genomic DNA was extracted using a DNA extraction kit from Promega (United States). The extracted DNA was detected using a NanoDrop ND-2000C spectrophotometer (Thermo Fisher Scientific, United States).

### Genotyping

**FIGURE 2:**
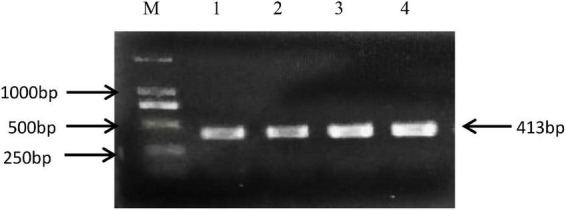
Electrophoresis of PCR amplification products of miR-181 c/d gene locus rs8108402. M, DNA marker; Lanes 1 to 4, PCR amplification products.

**FIGURE 3:**
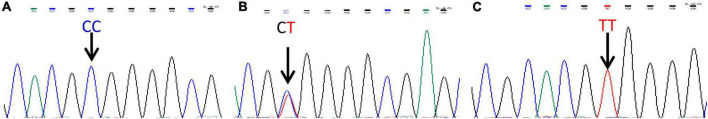
miR-181 c/d gene locus rs8108402 sequencing map. **(A)** CC genotype. **(B)** CT genotype. **(C)** TT genotype.

The rs8108402 C/T polymorphism was detected using polymerase chain reaction-sequencing-based typing. The base sequence of the miR-181c gene was queried from the GenBank of NCBI PubMed. Primers specific for polymerase chain reaction (PCR) amplification of the rs8108402 C/T polymorphism were designed using Primer Premier 5.0, as follows: upstream primer: 5′-CTCTGTGCGATCACTGGAGG-3′; downstream primer: 5′-GAAGTCTGGCAACGGAGGAT-3′. The primers were synthesized by Shanghai Biosun Sci&Tech Co., Ltd. The 25-μl reaction mixture consisted of 1 μl of prepared template DNA, 1 μl of upstream primer, 1 μl of downstream primer, 10 μl of 2 × Master Mix, and 12 μl of deionized water. The reaction was performed on a thermal cycler 9700 (Applied Biosystems, Foster City, CA, United States) with the following thermal parameters: pre-denaturation at 98°C for 3 min, followed by 35 cycles of denaturation at 98°C for 10 s, annealing at 59°C for 10 s, and extension at 72°C for 30 s, with a final extension step at 72°C for 10 min. The amplification products were electrophoresed on a 1.5% agarose gel, visualized by ethidium bromide staining (Figure 2), and subsequently sent to Shanghai Biosun Sci&Tech for sequencing. Sequencing results were analyzed using Chromas software (Version 2.6.5; Technelysium Pty Ltd., Australia) (Figure 3).

### Statistical analysis

All data were analyzed using SPSS23.0 (SPSS Inc., Chicago, IL, United States). The differences in the miR-181c/d gene locus rs8108402 genotype and allele frequencies between the KD and control groups, the KD-IVIG and KD-nIVIG groups, and the KD-CAL and KD-nCAL groups were evaluated using the *χ^2^* test. The Hardy-Weinberg equilibrium was assessed using the *χ^2^* test with 1 degree of freedom. Raw and adjusted odds ratios (OR) and 95% confidence intervals (CI) were subsequently calculated using univariate and binary logistic regression models to assess the associations of alleles and genotypes with KD susceptibility, IVIG unresponsiveness, and CAL risk. Statistical significance was set at *P* < 0.05.

## Results

### Amplification results of gene polymorphism

The 413 bp amplified fragment of the miR-181c/d gene locus rs8108402 was separated using 1.5% gel electrophoresis.

### Association of the miR-181c/d gene locus rs8108402 polymorphism with susceptibility to Kawasaki disease, intravenous immunoglobulin responsiveness, and coronary artery lesions formation

There were statistically significant differences in C and T allele frequency distributions between the KD group and healthy controls for the polymorphic site rs8108402 C/T (*P* = 0.002). The distribution of the genotypes CC, CT, and TT also presented statistical significant difference between the KD and control groups (*P* = 0.003). Compared to the rs8108402 C allele, the T allele was significantly associated with increased KD susceptibility (OR = 2.080, 95% CI = 1.317∼3.283) (Table 1). However, there were no significant associations discovered between the rs8108402 C/T polymorphism and IVIG unresponsiveness risk in the KD group (Table 2). Association of the rs8108402 C/T polymorphism with risk of CAL formation were also not statistically significant (Table 3).

**TABLE 1:** Genotype and allele frequencies of miR-181 c/d gene locus rs8108402 for the KD and control groups (%).

Alleles/ genotypes	KD	Control	*χ^2^*	*P*	OR (95%CI)
C	133 (66.5)	161 (80.5)	10.063	0.002	0.481 (0.305∼0.759)
T	67 (33.5)	39 (19.5)			2.080 (1.317∼3.283)
CC	40 (40.0)	63 (63.0)	11.596	0.003	0.392 (0.221∼0.692)
CT	53 (53.0)	35 (35.0)			2.094 (1.186∼3.697)
TT	7 (7.0)	2 (2.0)			3.688 (0.747∼18.211)

**TABLE 2:** Genotype and allele frequencies of miR-181 c/d gene locus rs8108402 for the KD-IVIG and KD-nIVIG groups (%).

Alleles/genotypes	KD-IVIG	KD-nIVIG	*χ^2^*	*P*
C	116 (67.4)	17 (60.7)	0.484	0.520
T	56 (32.6)	11 (39.3)		
CC	35 (40.7)	5 (35.7)	1.337	0.513
CT	46 (53.5)	7 (50.0)		
TT	5 (5.8)	2 (14.3)		

**TABLE 3:** Genotype and allele frequencies of miR-181 c/d gene locus rs8108402 for the KD-nCAL and KD-CAL groups (%).

Alleles/genotypes	KD-CAL	KD-nCAL	*χ^2^*	*P*
C	30 (62.5)	103 (67.8)	0.454	0.599
T	18 (37.5)	49 (32.2)		
CC	8 (33.3)	32 (42.1)	0.601	0.741
CT	14 (58.4)	39 (51.3)		
TT	2 (8.3)	5 (6.6)		

## Discussion

Kawasaki disease is a pediatric healthcare burden worldwide due to concerns of cardiovascular sequelae. In the United States, the prevalence of KD in ethnic East Asians is more than 2.5 times higher than that in Caucasians (14). IVIG and other therapies designed to modulate inflammation can effectively prevent the development of detrimental coronary aneurysms (15). Decreased cytokine and chemokine levels, decreased numbers of activated T cells, and increased numbers of circulating NK cells have been observed in patients with KD receiving IVIG therapy (16). In patients resistant to initial IVIG treatment, levels of inflammatory cytokines such as TNF-α, IL-6, IL-8, IL-17, IFN-γ, G-CSF, MCP-1, and SIL-2Rα, and anti-inflammatory cytokines such as IL-10, sTNFR1, and sTNFR2, were found to be simultaneously elevated (17), suggesting that a more thorough understanding of the pathophysiology of KD and cytokine networks is required for elucidation of possible pathogenic elements and development of more effective therapies. Our study examined the association between risk of IVIG unresponsiveness and the rs8108402 C/T polymorphism and discovered no statistical significance. Previous research by Onouchi et al. (18) has reported the association of ITPKC and CASP3 polymorphisms and risks for IVIG unresponsiveness and coronary artery lesion. However, studies examining the genetic basis of IVIG unresponsiveness in KD are still few and far between. More research is desirable to uncover the genetic and physiopathological mechanism of IVIG unresponsiveness.

Our study has discovered that the miR-181c/d gene locus rs8108402 C/T SNP is significantly associated with KD susceptibility risk in Chinese children, indicating a possible role for miRNAs in the pathogenesis and genetics of KD. Previously, dysregulation of numerous miRNAs in immunologic, epithelial-mesenchymal transition, and cell apoptosis signaling pathways have been identified in patients with KD (19). The miR-181 family is involved in various biological processes, including immunity, apoptosis, autophagy, cell proliferation, and mitochondrial functioning (6), for which possible altered expression by SNPs may contribute to KD progression through a variety of physiological pathways. One study discovered that miR-181c promotes TGF-β–induced differentiation of Th17 cells by targeting Smad7 and enhancing Smad2/3 signaling (20). Th17 cells secrete IL-17 and function in effective mucosal defense against certain pathogens and are associated with autoimmune diseases, including multiple sclerosis, Crohn’s disease, and psoriasis. In the vascular endothelium, IL-17 induces a strong pro-inflammatory response by stimulating the secretion of IL-6, granulocyte macrophage colony-stimulating factor (GM-CSF), vascular endothelial growth factor (VEGF), and C-X-C motif chemokine ligand 1/3/5/6/8 (CXCL1/3/5/6/8) to recruit neutrophils and T cells (21). It is of note that Th17 cell frequency and Th17 cell-associated cytokines were increased during acute KD and decreased after effective treatment.

Moreover, miR-181d targets the NF-κB pathway and promotes the expression of pro-inflammatory cytokine TNF-α (10), which is a pleiotropic inflammatory cytokine classically elevated during KD acute phase. Increased serum exosomal miR-181d is also associated with enhanced levels of IL-6 through possible mechanisms of targeting the suppressor of cytokine signaling 3 (SOCS3) (8). IL-6 stimulates the expression of acute phase reactants, promotes Th17 cell differentiation, downregulates Treg differentiation, increases VEGF production to increase angiogenesis and vascular permeability. IL-6 is upregulated during the acute stage of KD and is a possible biomarker for incomplete and IVIG non-responsive KD (22).

Our study discovered the association of KD with the SNP rs8108402 C/T, which is also located in the Nanos3 gene. Nanos3 is a functional transcriptional factor, and its polymorphism is associated with endothelial-mesenchymal transition (EMT) (11). EMT is involved in myofibroblast-like cell mediated damage to the coronary arterial wall in KD patients (11). One study found that EMT can be activated by sera from patients with KD by the KLF4-miR-483 axis in an miRNA–dependent pathway (23). Nanos3 and its polymorphisms could have associations with KD disease progression via EMT-associated mechanisms.

The miR-181c/d gene locus rs8108402 C/T is located in the promoter region of miR-181c and miR-181d genes and the intron of the Nanos3 gene and has been previously found to be associated with susceptibility to systemic lupus erythematosus (12). Transcription factor binding analysis demonstrated rs8108402 to be the binding site of possible Sp1 transcription factors, indicating its possible role in the transcriptional regulation of miR181c, miR181d, NANOS3 mRNA. Our study discovered that Chinese children who are carriers of the CT genotype and T allele are at a statistically increased risk of developing KD. However, no significant association was observed between the CAL and nCAL groups or the IVIG and nIVIG groups. More research, which may include direct genetic manipulations of animal models, is required to further elucidate the detailed biological and molecular mechanisms underlying of our findings. The limitations of our study include a relatively small sample size, for which a larger-scale study is desirable to verify the association between polymorphisms of miR-181 c/d/Nanos3 and risks of KD susceptibility, IVIG unresponsiveness, and CAL formation.

## Data availability statement

The original contributions presented in the study are included in the article/supplementary material, further inquiries can be directed to the corresponding author.

## Ethics statement

The studies involving human participants were reviewed and approved by the Ethics Review Committee and Institutional Review Board of the Third Xiangya Hospital. Written informed consent to participate in this study was provided by the participants’ legal guardian/next of kin.

## Author contributions

All authors listed have made a substantial, direct, and intellectual contribution to the work, and approved it for publication.

## Footnotes

1
http://gene-regulation.com
